# On Effectiveness and Legitimacy of ‘Shaming’ as a Strategy for Combatting Climate Change

**DOI:** 10.1007/s11948-017-9909-z

**Published:** 2017-04-11

**Authors:** Behnam Taebi, Azar Safari

**Affiliations:** 1 Department of Values, Technology and Innovation, Faculty of Technology, Policy and Management, Delft University of Technology, Delft, The Netherlands; 2 Belfer Center for Science and International Affairs, Kennedy School of Government, Harvard University, Cambridge, MA USA; 3 Faculty of Law, Leiden University, Leiden, The Netherlands

**Keywords:** Climate change, INDC, Naming and shaming, Multinational corporations, Corporate social responsibility, Environmental management

## Abstract

While states have agreed to substantial reduction of emissions in the Paris Agreement, the success of the Agreement strongly depends on the cooperation of large Multinational Corporations. Short of legal obligations, we discuss the effectiveness and moral legitimacy of voluntary approaches based on naming and shaming. We argue that effectiveness and legitimacy are closely tied together; as voluntary approaches are the only alternative to legally imposed duties, they are most morally defensible particularly if they would be the most effective in reducing the harmful greenhouse gases. Shaming could be made effective if states could prompt more corporations to accept voluntary cuts with high gains—such as public acknowledgements—and high losses, such as reporting on noncompliance and public exposure (naming), along with some kind of condemnation (shaming). An important challenge of such voluntary approaches is how to ensure compliance with the agreed upon commitments, while avoiding greenwashing or selective disclosure. Certain institutional arrangements are inevitable, including an independent measurement, monitoring and verification mechanism. In this paper, we discuss the potentials and ethical pitfalls of shaming as a strategy when corporations have a direct relationship with consumers, but also when they are in a relationship with governments and other corporations.

## Introduction: Whose Actions, Whose Obligations?

The Paris Agreement at the Conference of Parties (COP21) to the United Nations Framework Convention on Climate Change (UNFCCC) marks a historic turn in climate change policy. Among other changes, states have agreed to substantially reduce their greenhouse gases (GHGs) in order to limit global warming to 2 °C above pre-industrial levels. This agreement is based on accepting comprehensive national climate plans, or Intended Nationally Determined Contributions (INDCs), as the Paris agreement calls them. In achieving this level of GHG cuts, not only national states but also non-state actors such as municipalities and large corporations will play a crucial role. This could be problematic, because according to customary international law,^1^ states may only be held responsible for the actions and omissions of their official organs. So states, as the main parties under obligation, are making promises that will be more or less effective depending on the active participation of many non-state actors. This paper will focus on the role of one particular group of non-state actors, namely corporations—particularly large Multinational Corporations (MNCs), since they will play an essential role in making the global GHG cuts and making good on the national pledges.

One might argue that it is an ideal to introduce legally enforceable obligations for corporations. Assuming that “[a]voiding severe global catastrophe is a moral and legal imperative,” a group of legal scholars and practitioners has published the Oslo Principles, in which they argue that “all States and corporations have an immediate moral and legal duty to prevent the deleterious effects of climate change” (Spier et al. 2015, 1). Climate negotiations during the last two decades have dealt with the extent of the state’s legal duties. Corporations’ moral and legal duties are a much less discussed subject. The authors of the Oslo Principles have explicitly included corporations as the second entity (in addition to states) because of their *ability* to accomplish the needed GHG reductions to avert climate change.^2^ It is particularly relevant to consider the duties of these two most *capable* parties—namely states and corporations—in conjunction, because there is a relevant interaction between the two; i.e. states could play an important role in imposing certain restriction on corporations (legally binding or otherwise). Some scholars argue that corporations’ (legal) obligations could best be conceived as a direct corporate responsibility under the auspices of international law (e.g. Adeyeye 2007) but this is not the chosen model for reducing GHGs internationally, given that the Paris Agreement is an international accord with nation states as the only parties. Alternatively, states could impose legal restrictions on corporations in accordance with international agreements. This is to an extent the current practice, but as we will discuss in section “The Inadequacy of the Current Approach: States’ Unwillingness and Inability”, it has some serious shortcomings; i.e. states might be unwilling and sometime even unable to take action with respect to MNCs; the phenomenon of the “race to the bottom” is discussed in section “The Inadequacy of the Current Approach: States’ Unwillingness and Inability” as well.

Short of effective legally enforceable rules, we need to explore alternatives such as incentivizing voluntary commitments among corporations along with (non-compulsory) compliance mechanisms. In this respect, it is worthwhile considering “naming and shaming” as one of the key strategies for penalizing noncompliance in voluntary approaches. In a sense, naming and shaming is implicitly the strategy for ensuring compliance among states as well. During the negotiations prior to the Paris conference, it became clear that a legally binding agreement would not have much chance of political success. The Obama administration, for instance, decided to forego a binding treaty, since such a treaty could not count on the required two-thirds majority in the U.S. Senate; the administration’s negotiators therefore explicitly promoted a strategy to “name and shame” states into cutting their emissions (Davenport 2014).^3^ So the Paris Agreement is based on accepting pledges by the parties (i.e. nation states) and regularly reviewing those pledges with “negative reputational consequences” for countries that fail to meet their targets (Jacquet and Jamieson 2016, 645). Acknowledging that reputational effects would have an impact beyond individuals,^4^ Jacquet and Jamieson (2016, 643) suggest that the Paris Agreement could only succeed if ‘pledge and review’ will be carried out beyond the nation states with “the power of shaming laggards”. In this paper we investigate the effectiveness and the moral legitimacy of *shaming* as a strategy for incentivizing emission cuts among a very important group of non-state actors, namely large multinational corporations.

Indeed, there are already various mechanisms in place to incentivize and ensure non-state actors’ contributions to cutting GHGs. Many non-state actors, including a considerable number of large corporations, have already committed themselves to several emission cuts. These cuts are, however, voluntary commitments without any external verification or compliance assurance. For such voluntary approaches to be effective, there are at least two requirements that need to be met. First, there should be an incentive mechanism to prompt more corporations to accept such voluntary emission cuts, because the success of the agreement very much relies on the participation of a large number of MNCs. Such an incentive mechanism should have high gains—such as public acknowledgement of a corporation’s role in combatting climate change—and high losses, such as clear reporting on noncompliance and public exposure (naming), along with some kind of condemnation of such behaviour (shaming) (building on Friman 2015b). Second, since reporting on voluntary cuts is often based on self-assessments, there needs to be a verification mechanism in order to ensure compliance with the agreed-upon cuts. Section “The Role of Non-state Actors in Global Emission Cuts” focuses on specifying the non-state actors and their roles in combatting climate change. Section “Effectiveness of Shaming as a Strategy: Governmental and Non-governmental Approaches” presents several *quasi*-*judicial* 5 (inter)governmental and non-governmental experiences with similar issues. By drawing comparisons with Corporate Social Responsibility (CSR) and with agreements to voluntarily commit to environmental programs supported by intergovernmental and non-state actors, we investigate the extent to which “naming and shaming” could be a viable strategy for securing the contributions of MNCs for combatting climate change.

Section “Moral Legitimacy of Shaming as a Strategy” deals with the issue of moral legitimacy of such commitment based voluntary approaches. One might argue that approaches based on voluntary commitments (and shaming the one who would not comply) might blur the fundamental moral obligations and are, therefore, ethically illegitimate. In this section, we will investigate the nature of moral obligations of corporations and the legitimacy of replacing such obligations with voluntary based approaches such as the one presented in this paper. We argue that the effectiveness and legitimacy questions are closely tied together; provided that such voluntary based (quasi-judicial) approaches are the only alternative to legally imposed duties, they are most morally defensible if they would indeed be most effective in reducing the harmful GHGs. Section “Moral Legitimacy of Shaming as a Strategy” further discusses the ethical limitations and pitfalls of voluntary approaches. Section “Discussions and Conclusions: Under Certain Conditions, Shaming Could Work” presents our conclusions.

## The Inadequacy of the Current Approach: States’ Unwillingness and Inability

As mentioned in the introduction, imposing legal restriction by nation states on MNCs might be rather problematic. One of the most significant challenges is the so-called “race to the bottom.” That is, when host states look for more investment-driven development, they are willing to barter their power of regulation in exchange for short-term economic gains; “[i]n order to attract investment, many nations, particularly developing ones, will acquiesce to a corporation’s needs … [by establishing] a corporate-friendly legal environment” (Macek 2002, 104). To be more precise, states seem to be reluctant to impose limits on MNCs, thereby sacrificing their national interests in order to comply with international obligations. This race to the bottom often compels these states to lower their human rights standards, especially where labour rights are concerned, and to deregulate environmental and tax laws (Deva 2003, 2004; Holland 2010; Milanovic 2009).

In addition to being unwilling, some states rely on the revenues from MNCs to such an extent that they cannot afford to lose those revenues by imposing restrictive regulations. Hence, when a state’s revenues are consolidated heavily in one entity, that state might simply be *unable* to impose restrictions on MNCs; a race to the bottom is then inevitable (Revak 2012). In a study performed in 2000, Anderson and Cavanagh showed that among the 100 biggest “economies” in the world, 51 are corporations and 49 are countries (Anderson and Cavanagh 2000).^6^ Moreover, “[t]he annual revenues of General Motors are greater than the GDP of more than 148 countries; while Wal-Mart’s revenues exceed the combined GDP of sub-Saharan Africa, excluding South Africa and Nigeria” (Stiglitz 2007, 476). Indeed, that this is the case that these are large and powerful companies does not necessarily imply that they are unwilling to take responsibility for their environmental and climate related impact. Wal-Mart was one of nine massive companies that announced prior to the Paris negotiations that they would switch to 100% renewable energy; this was part of the RE100 Campaign as it will be discussed in section “The Role of Non-state Actors in Global Emission Cuts”. Many corporations seem willing to contribute to global GHG cuts, because they acknowledge the importance of avoiding catastrophic climate change and also because they see long-term returns on their low carbon investments. But it should be mentioned that these nine corporations also epitomize the problem at hand; they have received extensive media exposure because they are on the list of Fortune 500 companies, meaning that only nine out of these 500 have pledged substantial action. Moreover, making pledges and commitments is one thing, but acting upon these promises and having the results externally audited is another. This issue is discussed at length in the following sections.

## The Role of Non-state Actors in Global Emission Cuts

Since the Copenhagen Conference of Parties in 2009, there has been a new discourse surrounding climate change, focusing on limiting the increase of the global temperature above certain levels. The 2 °C increase compared to pre-industrial levels was a limit agreed upon in Copenhagen; the Paris Agreement reiterated this target and made this goal more ambitious by presenting the more stringent target of 1.5 °C above pre-industrial levels, “recognizing that this would significantly reduce the risks and impacts of climate change” (UNFCCC 2015, Article 2). In line with the Copenhagen Agreement, recent policies regarding the target emission are, therefore, expressed in terms of an *emission gap* between business-as-usual and the desirable emission cuts that could help to meet the 2 °C target. This has proven to be a helpful approach, since it shows that continuing the business-as-usual scenario would be utterly insufficient for meeting the target, as demonstrated by the United Nations Environment Program (UNEP) “Bridging the Emission Gap” annual reports. The UNEP report from 2011 stressed that there is a need to cut 12 gigatons of CO_2_ equivalent (Gt CO_2_e)^7^ globally, while national government pledges back then indicated a willingness to cut only half that amount (UNEP 2011). The Paris Agreement—if it proves successful—aims at substantially narrowing this gap in order to ensure that the global temperature would not rise above the indicated 2 °C.^8^

Prior to this new thinking about emission cuts, scenarios seemed plausible in which “sovereign national governments agree under the UNFCCC on emission reductions; they subsequently introduce in their jurisdiction the right incentives for emission reductions; and finally, companies, municipalities, other organizations and individual citizens take measures to reduce their greenhouse-gas emissions” (Blok et al. 2012, 471). This top-down approach was, however, deemed insufficient. A new type of climate policy was needed, based on a combination of top-down and bottom-up approaches, in which non-state actors would also play a central role (Blok et al. 2012; Hsu et al. 2015; Jacquet and Jamieson 2016). This need was acknowledged by the introduction of the Non-state Actor Zone for Climate Action (NAZCA) at the Lima conference in 2014.^9^ Likewise, the Paris Agreement welcomed “the efforts of non-Party stakeholders to scale up their climate actions, and *encourages* the registration of those actions in the Non-State Actor Zone for Climate Action platform” (UNFCCC 2015, 17 emphasis in original).

The term “non-state actor” requires some explanation here. Strictly speaking, non-state actors are those actors that are not parties to the UNFCCC. Where climate change is concerned, there are various subnational and supranational non-state actors, including municipalities, regions, national and international non-governmental organizations (such as environmental organizations), and corporations. This paper focuses on large corporations that have a crucial role to play in cutting greenhouse gases. There are already a large number of International Cooperative Initiatives (ICIs) that report on non-state actors’ efforts to reduce climate change. The “Bridging the Emission Gap” publications report on these and other non-state actors’ initiatives for global mitigation efforts.^10^ Thirty such initiatives are currently listed on the Climate Initiatives Platform, “a new online portal for collecting, sharing and tracking information about International Climate Initiatives” (UNEP 2015).^11^ Many large corporations have thus already committed themselves to voluntary GHG cuts in the coming years. Prominent initiatives include the Business Environmental Leadership Council (BELC), which is “the largest US-based group of corporations”; the Caring for Climate Initiative, which aims to advance the role of businesses in climate change; and RE100, which wants “at least 100 companies to make a global 100% renewable commitment” within a reasonable time frame (UNEP 2015, 41).

The current approach to corporations with respect to climate change has two main problems. First, a large number of the currently operable corporations that could have serious impact on GHG cuts are essentially MNCs, operating in different nation-states. As discussed in section “The Inadequacy of the Current Approach: States’ Unwillingness and Inability”, imposing legal restrictions by individual states might be rather problematic and sometimes simply impossible. Second, all the aforementioned initiatives are based on the voluntary commitments of corporations or groups of corporations; reporting is mostly based on self-assessment and is often without third-party verification. Section “Effectiveness of Shaming as a Strategy: Governmental and Non-governmental Approaches” elaborates on this shortcoming of the existing mechanisms, by comparing it to similar situations in other areas.

## Effectiveness of Shaming as a Strategy: Governmental and Non-governmental Approaches

As mentioned earlier, countries have agreed to certain emission cuts—or the INDCs—in the Paris Agreement. While the detailed implementation of these INDCs remains unclear, it seems likely that their *measuring, reporting and verification* (MRV) will not be in the hands of state actors alone. Earlier international negotiations (most notably in Bali and Cancun) have established mechanisms for MRV procedures. The international verification of national reports should take place through international review, “which is a process to increase the transparency of mitigation actions and their effects, and support needed” (UNFCCC 2014, 16). It seems clear that transparency and trust are the key issues in international reporting and verification, as has been emphasized in Article 13 of the Paris Agreement: parties need to build “mutual trust and confidence and to promote effective implementation, an enhanced transparency framework for action and support” (UNFCCC 2015, 28). Transparency and trust are also key to the domestic implementation of the Agreement within the states, and for the reporting and verification of the reductions as promised by non-state actors, including MNCs.^12^ In this section, we first review some quasi-judicial experiences with similar issues, such as Corporate Social Responsibility (CSR), environmental management and sustainability. We then discuss the requirements for an effective naming and shaming strategy, also considering empirical evidence from the literature (Friman 2015a).

In the absence of international or domestic legally-binding responsibilities (also known as hard law) for MNCs, several internationally-recognized intergovernmental and non-governmental organizations have been formed within the realm of soft law. Soft law is a quasi-legal instrument that aims to institutionalize a social norm without exerting legally-binding force. Most efforts in this area have been devoted to addressing transparency and disclosure; they aim to create incentives for MNCs to behave ethically sound, specifically where human rights and CSR are concerned (Sutton 2003; Backer 2008). Two important instances of state-involved soft-law innovations are the initiatives of the Organisation for Economic Co-operation and Development (OECD) and the United Nations Global Compact (UNGC). The OECD has developed principles that build on the notion of transparency, including the OECD Principles of Corporate Governance (OECD 2004) and the Guidelines for Multinational Corporations (OECD 2008). These provide “the contours for a system of monitoring and reporting that have a potentially significant application to issues of environmental transparency […] without invoking the formal structures of the domestic legal orders of participating states” (Backer 2012, 110). The UNGC is perhaps one of the most recognizable initiatives for the collection and disclosure of CSR-related information (Akhtarkhavari 2010); it is organized around ten conduct-oriented principles covering subjects such as “human rights, labour, environmental and anticorruption values,” all of which aim to create a framework for corporate accountability (Backer 2012, 115). Though the UNGC requires an Annual Communication of Progress (CoP), this communication is based on self-reporting. Nonetheless, failing to comply could change an organization’s status to ‘non-communicating’ or ‘inactive.’

Among the non-governmental initiatives, the Global Reporting Initiative (GRI), the International Organization of Standardization (ISO) and the Carbon Disclosure Project (CDP) are examples of organizations with huge international credit and publicity power. The GRI is a non-profit that helps corporations and other organizations “understand and communicate the impact of business on critical sustainability issues such as climate change, human rights, corruption and many others.”^13^ Compared to the UNGC, the GRI demands a greater level of detail in its requests for disclosure and has much less media appeal; all the same, it has produced a successful and often used disclosure mechanism (e.g. Hedberg and von Malmborg 2003; Brown, de Jong, and Lessidrenska 2009; Backer 2012). The ISO is mainly involved in developing standards for environmental transparency and for communication about environmental management; among other things, ISO provides for the most widely adopted voluntary environmental programs (Matthews 2003; Prakash and Potoski 2006; Backer 2012). Finally, the CDP is also a non-profit organization that provides reporting mechanisms. CDP’s website explains that one of the benefits of becoming a signatory is “public recognition of your commitment to engaging with companies on issues of climate change.”^14^ Companies self-report to CDP by means of a questionnaire, indicating whether their report and assessment have been verified or whether such verification is still underway (UNEP 2015, 40). However, like many other voluntary initiatives, third-party verification does not seem to be standard practice.

What all the aforementioned intergovernmental and non-governmental mechanisms share is that they work based on voluntary commitments. Going back to the main focus of this paper, we argue that an approach based on the voluntary engagement of MNCs in substantially reducing GHG emissions will only be successful if (i) there are incentive mechanisms for motivating more corporations to accept such voluntary emission cuts and (ii) there is an independent monitoring and verification system. Let us elaborate on these two requirements.

An incentive mechanism should have high gains, such as public acknowledgement of the responsibility a corporation assumes for combatting climate change, such as the extensive media exposure that Wal-Mart and other eight Fortune 500 companies were receiving because they are moving toward to 100% renewable energy consumption. More broadly, accepting voluntary emission cuts, for instance by becoming a member of influential and credible international organizations such as the UNGC and CDP, has mostly reputational benefits for corporations. But there should also be high losses, such as clear reporting on noncompliance and public exposure (naming), as well as condemnation of such behaviour (shaming). The question is whether such naming and shaming could be a potent deterrence strategy and if so, under what conditions. Let us elaborate on some key element of a system of naming and shaming as a policy instrument that could potentially maximize the impact on corporations’ compliance. Building on Friman (2015a), 203, we distinguish between the three key steps of naming or “the public identification of noncompliance,” shaming or “public condemnation of such behaviour” and some kind of “material sanction” that could strengthen shaming.^15^ Let us review these three steps in more detail.

An effective naming strategy hinges on the *credibility* of the organization that could name noncompliance and the *reliability* of the information that substantiates the naming. If states would be the parties that would *name* the noncommitting corporations, the issue of credibility does not seem to be a problem here.^16^ Things become more complicated when it comes to the reliability of the information, since in a voluntary approach the information is based on self-assessments of corporations; a robust verification mechanism based one external audits seems to be indispensable here. There are two potential problems here. First, if we want shaming—as the next step that must follow naming—to be an effective strategy, the results of the external audits must become public. This could, in turn, make it more difficult for corporations to commit to such external audits. The relevant literature shows that corporations submit more readily to external audits if they know that the reports are not going to be public (e.g. Potoski and Prakash 2004). This gives rise to a dilemma between making shaming more effective on the one hand and increasing corporations’ participation on the other. Second, there is the issue of *selective disclosure*, as defined by Marquis et al. (Forthcoming) as “a symbolic strategy whereby firms seek to gain or maintain legitimacy by disproportionately revealing beneficial or relatively benign performance indicators to obscure their less impressive overall performance.” This problem emphasizes the need for clear and unified measurement, monitoring and verification practices, such that the final outcome could rule out selective disclosure. In sum, for naming to be possible, a robust measurement, monitoring and verification mechanism is needed to ensure the reliability and quality of the information provided in corporations’ self-assessments.

After the disclosure of the information about noncompliance, the next step is to move to shaming or condemnation of such an act. At first glance, being labelled noncompliant might seem to have a negative impact on corporations. Media can shape public opinion by providing people with evidence of such noncompliance. In most CSR cases known in the literature, after shaming MNCs have tried to showcase their collaboration with the international community by accepting public criticism and employing redressing measures to become compliant. At times, they have attempted to compensate for such violations by enhancing the support for consumers and workforces. Two interesting examples are worth mentioning here. The first is Nike that was involved in labour violations and other exploitative employment practices in the late 1990s. The company was then subjected to massive public disapproval which resulted in loss of profit and reputation. Nike responded to this by accepting the critique and implementing a fairly strict supply chain (in accordance with CSR) (Waller and Conaway 2011; Shavers 2012).^17^ The second example of successful shaming is when Shell proposed the disposal of the Brent Spar oil platform in the deep sea in North Sea; the proposal involved letting the platform sink rather than to dismantle and dispose of it onshore. Greenpeace started a powerful campaign resulting in, among other things, a widespread boycott of Shell Gas Stations in Northern Europe, which in turn let Shell abandon the plan (Gunningham 2009; Rosen-Zvi 2011).^18^

However, most of the known examples of when shaming has proven to be effective concern corporations that are in direct contact with their consumers; damage to their image could therefore directly undermine their business with individual consumers. Instead of such a Business-to-Consumer (B2C) relationship, many large corporations are engaged in Business-to-Business (B2B) or Business-to-Government (B2G) relationships, in which individual consumers’ pressure is less and sometimes non-existing. Indeed, in a B2B and B2G settings shaming could still happen as a result of one corporation shaming the other corporation (as in B2B) or a government shaming a corporation (as in B2G). In a B2G situation the impact of shaming seems evident. It seems, however, unlikely that governments will shame corporations (in a B2G relation) because, first, the ‘race to the bottom’ problem could play a role in state-corporations interaction and, second, it neglects the de facto inability of economically less powerful states to impose restrictions on MNCs or to shame those powerful MNCs for not complying with commitments. The most problematic situation is the B2B relations in which neither the reputation damage nor a direct relation with the state would be a deterrent.

In sum, we could say that shaming could be an effective strategy for incentivizing corporations to cut GHG emissions if (i) there would be high benefits associated with accepting those commitment (such as public acknowledgement of the responsibility a corporation) and high costs associated with non-compliance and (ii) there would be an international independent measurement, monitoring and verification mechanism in place. Moreover, the literature suggests that shaming would be potentially most powerful in B2C relationships. In B2G relationships shaming could lose power (depending on the willingness and ability of the states); in B2B relationships shaming is most problematic as a strategy because, first, there is little public information about those interactions and, second, reputation damage is not a potential deterrent anymore.

## Moral Legitimacy of Shaming as a Strategy

Section “Effectiveness of Shaming as a Strategy: Governmental and Non-governmental Approaches” discussed shaming as a strategy for incentivizing emission cuts among large MNCs. In this section, we focus on the moral legitimacy of such approaches. One might argue that approaches based on voluntary commitments (and shaming the one who would not comply) might blur fundamental moral obligations and are, therefore, ethically illegitimate. In this section, we address the question of moral legitimacy by, first, focusing on the nature of moral obligations of corporations and, second, the legitimacy of replacing such obligations by voluntary based approaches such as the one presented in this paper.^19^

Many people acknowledge that corporations have special obligations when it comes to protecting the environment, but there is no consensus about the nature and the extent of such obligations. Defending market solutions, Bowie (2013), for instance, argues that corporations have an obligation to abide by law and environmental legislation.^20^ In his account, the moral responsibility for the environment lies mostly with consumers that could increase the demand by buying environmentally friendly products; the higher the demand, the more likely it becomes that corporations would act accordingly, as the argument goes. Against such minimalist account of corporations’ obligations, Arnold and Bustos (2005), Arnold (2013) defend a historical account of obligations; i.e. corporations that have contributed to the accumulation of the harmful GHGs have a moral obligation to deal with the consequences.^21^ So, they argue that large corporations (with massive energy consumption) have contributed to the existence and the perpetuation of global climate change. Bowies’ minimalist account of obligations further fails because it is based on a hidden (and mostly unsubstantiated) assumption, namely that all corporations are in B2C relations, that is, consumers are always in a direct relationship with corporations and, by that in the position to steer corporations’ policies by their purchases (or boycotts). This assumption is problematic for three reasons. First, as we have argued in section “Effectiveness of Shaming as a Strategy: Governmental and Non-governmental Approaches”, many corporations (especially bigger ones that are most responsible for GHGs) have a strong relation with governments (B2G) or with other corporations (B2B). So, there is not always a B2C relationship that could put consumers in the right position to exert their influence. Second, even when corporations are in B2C relations, the consumer does not always have full disclosure about the supply chain and the subcontractors of the main corporation. So, even if the consumer would be willing and able to steer a corporation’s behaviour, she does not always have access to information regarding the whole supply chain. Third, such emphasis on consumers presupposes that all consumers are in the luxurious position to be able to afford to choose between environmentally friendly and unfriendly products. This neglects the issue of poverty, not only in developing countries and emerging economies (with lower standards of well-being) but also in industrialized countries. In sum, shifting environmental responsibilities from the corporations to consumers is an unhelpful and problematic approach.

Another way of approaching (moral) obligations of corporations is based on their *ability*; i.e. one could also argue that corporations and nation states are the two most *capable* parties that could contribute to the mitigation of climate change by substantially reducing the GHGs. The Oslo Principles take this stance in defending moral and legal obligations for corporations (Spier et al. 2015). Assigning obligations based on both historical accountability and the capability of the parties has been intensely debated when discussing distributions of responsibilities among nation states; the United Nations Framework Convention on Climate Change (of which the most recent Conference of Parties meeting was held in Paris, leading to the Paris Agreement) is based on the notion of ‘common but differentiated responsibility.’ Many discussions in the field of climate ethics aim at spelling out the extent of these obligations for different states. It is not our intention to reiterate those arguments here,^22^ but it is essentially the same rationale that could be expanded to include the role of corporations that have contributed to the existence of the problem on the one hand, and could contribute to its solution, on the other.

Let us now turn to the question of the moral legitimacy of replacing moral obligations with voluntary based approaches. If we agree that there are certain moral obligations for corporations, the most straightforward action would be to turn those obligations into legal duties. As mentioned in section “Introduction: Whose Actions, Whose Obligations?”, the duties of corporations can be seen either as a direct corporate responsibility under the auspices of international law or as duties that national states must impose on corporations. The international approach is not the chosen model in the Paris Agreement, and it is probably going to be difficult to include an international enforcement mechanism. Imposing legal duties by nation states might be rather problematic too, because states might be unwilling or sometime unable to impose such restriction (as discussed in section “The Inadequacy of the Current Approach: States’ Unwillingness and Inability”). So, an alternative approach is a mechanism of (voluntary) pledge and review. The Paris Agreement presents such an approach for dealing with states; i.e. countries commit to voluntary emission cuts and report on their progress; the laggards will then be shamed for being late or non-compliant. This is called by Jacquet and Jamieson (2016) the “soft but significant power” of the Paris Agreement. In this paper, we have extended the same rationale to include corporations in the analysis. Since climate change is probably the most complex problem the world is facing and humanity has not been successful is averting it so far, all actors must get motivated to get involved (Jamieson 2014). The top-down approaches in the pre-Paris era have not been successful. These voluntary bottom-up approaches seem to be the only feasible alternative to comprehensive legally binding duties. In addressing the question of moral legitimacy, we argue that the effectiveness and legitimacy are tied together, that is, assuming that such voluntary based (quasi-judicial) approaches are the only alternative to legally imposed duties, such approaches are most morally defensible if they would indeed be the most effective in reducing the harmful GHGs.

## Discussions and Conclusions: Under Certain Conditions, Shaming Could Work

Implementing the ambitious goals of the Paris Agreement depends on the contributions of both states and non-state actors, most notably large Multinational Corporations (MNCs). In the absence of relevant legally imposed regulations, national states might instead incentivize MNCs to engage in voluntary cooperation to cut their emission gases. An important challenge of such voluntary approaches is how to ensure compliance with the agreed upon commitments. In this paper we have investigated the effectiveness and the moral legitimacy of *shaming* as an approach for penalizing noncompliance. Let us first start by addressing the question of moral legitimacy. One might argue that having legally enforceable rules under the auspices of the international law would be the most defensible option, but unfortunately this is not the chosen practice in the Paris Agreement and it seems unlikely that such legally enforceable rules will be introduced any time soon. Assuming that voluntary based approaches are the only alternative to legally imposed duties, we have argued that such approaches are a morally defensible solution if they would indeed be most effective in reducing the harmful GHGs. In our argument, effectiveness and legitimacy are strongly interlinked. This brings us to the question of effectiveness.

While eliciting voluntary cooperation might sound too informal and noncommittal, and thus ineffective, there are good reasons to believe that such incentives would work. In section “Effectiveness of Shaming as a Strategy: Governmental and Non-governmental Approaches”, we have discussed several governmental and non-governmental initiatives that are quite successful in promoting Corporate Social Responsibility and incentivizing environmental management issues, despite a lack of legal enforcement mechanisms. A good example appears in one of the standards of the International Organization of Standardization (ISO) for environmental management: ISO14001 requires corporations to spend a good deal of money to create environmental management systems and yet, this is among the most widely applied standards (Prakash and Potoski 2006). In general, it seems fair to argue that voluntary based (non-binding) approaches could be effective too. As Rosen-Zvi (2011) correctly argues, the question is not *whether* voluntary based approaches are effective but *under which criteria* they could be effective. Likewise, we argue that under certain criteria shaming could be an effective strategy for incentivizing emission cuts by large corporations. Those conditions could best be formulated by discussing the ethical pitfalls of voluntary approaches. We will recapitulate several pitfalls as discussed in this paper, while discussing how they could best be responded to.

The first ethical pitfall is that voluntary commitments might shift the attention from fundamental moral obligations that corporations have in combatting climate change (as discussed in section “Moral Legitimacy of Shaming as a Strategy”) to “charity-based” commitments that corporations could make; this leads some corporations to choose to comply only selectively (Deva 2003). To be more precise, MNCs are free to choose among various regulations, to follow their desired implementation methods and to release only the information they are willing to disseminate. This is also referred to as *greenwashing* or selective disclosure, that is when corporations decide to reveal only beneficial or benign performance results (Marquis, Toffel, and Zhou Forthcoming). We have argued that certain institutional arrangements need to be put in place in order to reduce the risk of greenwashing. More specifically, we think that after corporations have agreed to certain emission cuts, there must be an independent measurement, monitoring and verification mechanism in order to ascertain that the volunteered cuts are real and not just empty commitments.

As mentioned earlier, the literature suggests that ‘naming and shaming’ could contribute to a behaviour change among corporations, but “the degree to which shame functions to change behaviour varies widely across firms and sectors” (Haufler 2015, 199). Likewise, we acknowledge that there is no one-size-fits-all for all corporations. More specifically, we zoom in on the different types of corporations’ relationships, being Business-to-Consumer (B2C), Business-to-Government (B2G) and Business-to-Business (B2B). Before discussing the pitfalls of each type of relationship, two remarks seem to be in order. First, we don’t claim that all corporations fall in one of the three categories; business corporations could be in a varying degree in business with direct consumers, the government or other corporations. Second, our analysis did not have the ambition to be comparative; we have merely focused on how shaming could serve as a strategy to incentivize good behaviour and to penalize non-compliance in each business relationship type and discussed the potential and the pitfalls of shaming as a strategy in each type of business relationship. Future empirical research needs to explore the effectiveness of shaming strategies in different business relationships and in different (industrialized and developing) states.

The most prominent examples in the literature, in which shaming has influenced corporations’ behaviour are in issues associated with CSR and environmental management. In these examples shaming has proven to be effective because those corporations were in direct contact with their consumer, or in a so-called B2C relationship. So, if shaming would be adopted as a strategy when corporations are voluntarily agreeing to emission cuts, those corporations that are in direct relationship with their individual consumers would be most likely sensitive to such a strategy. An important condition that needs to be met here is that there must be a reliable source of information about the corporations’ behaviour. So not only should there be an independent measurement and monitoring mechanism in place (pertaining to the first greenwashing pitfall) but also this information must be made public before shaming could be effective. This need for transparency, however, might cause some corporations to be less eager in accepting voluntary emission cuts in the first place, as discussed in section “Effectiveness of Shaming as a Strategy: Governmental and Non-governmental Approaches”.

When corporations have a relationship with states (in a B2G setting), the shaming mechanism works differently. States are in principle in a position to shame a corporation for lack of proper behaviour (or lack of compliance with the agreed upon cuts) but there are at least three types of pitfalls. First, states might be inclined to wrongly blame corporations, for instance to cover for their own incompetence or lack of compliance with the international agreements. Second, in economically less powerful states, there might be a serious reluctance to impose any restrictions on corporations or to shame corporations for non-compliance (as discussed in section “The Inadequacy of the Current Approach: States’ Unwillingness and Inability”). This problem could be resolved by involving the (stronger) state in which parent companies of the MNCs are active or by involving international organizations. Indeed, this is presupposing that economically more powerful states in which the parent companies of MNCs are active would be willing to cooperate. The latter might prove to be an unsubstantiated presupposition, as we will discuss later in this section. This brings us to the third pitfall of shaming in a B2G setting, that is, the race to the bottom. A possible solution to this problem (both in economically powerful and less powerful states) is that when corporations voluntarily commit to any emission cuts, these commitments will be publicly disclosed so that independent international organizations^23^ could be involved in the process of shaming. There are already several examples of such powerful international organizations that are involved in collecting independent and reliable data (as discussed in section “Effectiveness of Shaming as a Strategy: Governmental and Non-governmental Approaches”).

The third business type relationship is when corporations are in direct relation with each other. For shaming to be an effective strategy we need to assume that (i) corporations would be informed about each other’s voluntary commitments and (ii) willing to shame each other if one party does not comply. However, a lot of what is happening in B2B relationships is likely to stay outside of the public eye and public scrutiny. For shaming to be effective here, there needs to be a third overseeing party (either an international organization or a state) that could engage in shaming whenever needed. Of course, this is again presupposing that there is independent and reliable information available about the performances of each corporation.

To be sure, we do not claim to have found the silver bullet to ensure corporations’ contributions to combatting climate change. We fully acknowledge the problems with this approach mostly for incentivizing MNC action in the developing countries, but also in some industrialized nations where the governments do not fully support the Paris Agreement. For instance, some politicians in the U.S. have consistently denied the human induced impact of climate change and the findings of the IPCC; the recently elected President Donald Trump seems to strengthen these voices. If the U.S. decides to not comply with its INDC or to withdraw from the Agreement altogether, it could seriously weaken the Paris Agreement; i.e. the world powers, most notably the biggest polluters, seem to have kept each other in a prisoners’ dilemmatic balance in this agreement. Our argument rests on the assumption that the Paris Agreement will stay in place and that both states and non-state actors such as corporations will be engaged in making it a success.

## Footnotes

1 This has also been emphasized in the Draft Articles on Responsibility of States for Internationally Wrongful Acts, also known as the DARSIWA 2001 (ILC 2001).

2 While the Principles document does not elaborate on this issue, the commentary that accompanies this document explicitly discusses this issue; see page 64 in the following commentary document: http://www.osloprinciples.org/macmillan/globaljustice/Oslo%20Principles%20Commentary.pdf

3 In the international relations literature, naming and shaming has been thought to shape or change states’ behaviour. It is a strategy frequently used by human rights organizations; see, for instance, various contributions in (Friman 2015c). This paper, however, asks how shaming could incentivize corporations to comply with the targets set forth in the Paris Agreement.

4 It has been empirically shown that the threat of disapproval (i.e. shaming) could lead to more cooperation at the individual level; See (Jacquet et al. 2011; Jacquet 2015).

5 A quasi-judicial mechanism is essentially judicial in character but it is not within the officially defined judicial power as for instance defined in constitutions. An important feature of a quasi-judicial mechanism is that there is no need to have a *de facto* judge in the procedure, meaning that arbitrators or even scientific experts may perform as deciding authorities. Another important difference with judicial mechanism is that quasi-judiciary mechanisms often lack an enforcement mechanism; naming and shaming or other punitive actions such as suspension or termination of non-compliant parties' conventional rights seem to be the surrogate for actual legal enforcement. In section “Effectiveness of Shaming as a Strategy: Governmental and Non-governmental Approaches”, we discuss several examples of quasi-judicial mechanism in discussions on Corporate Social Responsibility and environmental management.

6 The comparison is based on countries’ GDPs and corporations’ sales.

7 This is a unit for expressing the amount of greenhouse gases. Different GHGs have different impacts on global warming—methane, for instance, is a much more potent GHG than CO_2_—but we need to be able to express everything in a single unit. A CO_2_ equivalent expresses all entities in an amount comparable to CO_2_.

8 According to Stavins (2015), 96% of global emissions is accounted for in the Paris Agreement, since “186 of the 195 members of the UNFCCC submitted INDCs by the end of the Paris talks.”

9 See the *Lima Call for Climate Action* Decision-/CP20 (UNFCCC 2014); http://unfccc.int/files/meetings/lima_dec_2014/application/pdf/auv_cop20_lima_call_for_climate_action.pdf.

10 See, for instance, Chapter 5 in (UNEP 2015).

11 Citation from the website of this initiative; see http://climateinitiativesplatform.org/index.php/Welcome. Consulted on 3 March 2016.

12 In more technical legal terms, in the international verification process the Biennial Update Reports (BURs) of each country will be reviewed in a procedure for International Consultation and Analysis (ICA). National verifications need to be organized domestically, and states need to report on their domestic verification in their BURs (UNFCCC 2014, 16).

13 Cited from the webpage of the GRI: https://www.globalreporting.org/information/about-gri/Pages/default.aspx. Consulted on 15 March 2016.

14 Adopted from the CDP website: https://www.cdp.net/en-US/Programmes/Pages/becoming-a-signatory.aspx. Consulted on 15 March 2016.

15 This is based on the synthesis analysis in the concluding chapter of the volume “The Politics of Leverage in International Relations: Name, Shame and Sanction” (Friman 2015c). While most contributions in this book are about the role of naming and shaming in international relations, the basic rationale of the arguments applies to the type of naming and shaming as discussed in this paper too.

16 We are assuming that states will not wrongfully ‘name’ a corporation, for instance in order to cover their own failure or incompetence. While this possibility exists, it seems unlikely that states would often undertake any ‘naming and shaming’ activity at all because of their unwillingness (race to the bottom) and inability (in case of less powerful states). So, the issue of the credibility of the organization (other than states) that does the ‘naming’ remains a concern.

17 For a detailed discussion of this case, see (Sutton 2003, 1170).

18 The Brent Spar case is a special case in that Shell seemed to have sufficient scientific evidence that their original proposal was the one that should have been preferred from an environmental point of view. Greenpeace suffered some reputation damage afterwards because their scientific evidence seemed to be flawed. We did include this example nonetheless because this case study seems to mark an important moment in which “major reputation sensitive companies” realized the power of shaming that could be “unleashed upon them by NGOs” (Gunningham 2009, 196).

19 Indeed, when fulfilling ethical obligations would become voluntary this would not loose their ethical stringency. However, when we agree that there are moral obligations but instead of turning those into legal obligations we leave the fulfilment of these moral obligations to voluntary approaches, it does require a reflection on the legitimacy of such voluntary approaches. This section deals with that question of legitimacy.

20 Environmental concerns and concerns for climate change are in principle distinct and only partly overlapping. Many authors, however, refer to them interchangeably, especially when it comes to questions of corporations’ responsibilities for dealing with these concerns.

21 This argument is based on the assumption that corporation would have been aware of such consequences. The authors argue that this is a reasonable assumption, certainly after the wide scientific acknowledgement of anthropogenic climate change in the beginning of this century by the IPCC.

22 Interested readers could consult two leading anthologies on these and other related climate ethics subjects with regard to national and international responsibilities (Gardiner et al. 2010; Arnold 2011).

23 Indeed, the same problems and issues might again play at the level of such organization. Questions such as how the organizations should be financed, to whom does it report, how does it handle matters that are revealed to it "in confidence" etc. are relevant questions that need to be addressed prior to initiating any international organization for this purpose.
